# Extraction, Analytical and Advanced Methods for Detection of Allura Red AC (E129) in Food and Beverages Products

**DOI:** 10.3389/fmicb.2016.00798

**Published:** 2016-05-27

**Authors:** Kobun Rovina, Shafiquzzaman Siddiquee, Sharifudin M. Shaarani

**Affiliations:** ^1^Biotechnology Research Institute, Universiti Malaysia SabahKota Kinabalu, Malaysia; ^2^Faculty of Food Science and Nutrition, Universiti Malaysia SabahKota Kinabalu, Malaysia

**Keywords:** Allura Red, toxicological, spectrophotometry, capillary electrophoresis, thin-layer chromatography, high performance liquid chromatography, electrochemical sensor, molecularly imprinted polymers

## Abstract

Allura Red AC (E129) is an azo dye that widely used in drinks, juices, bakery, meat, and sweets products. High consumption of Allura Red has claimed an adverse effects of human health including allergies, food intolerance, cancer, multiple sclerosis, attention deficit hyperactivity disorder, brain damage, nausea, cardiac disease and asthma due to the reaction of aromatic azo compounds (R = R′ = aromatic). Several countries have banned and strictly controlled the uses of Allura Red in food and beverage products. This review paper is critically summarized on the available analytical and advanced methods for determination of Allura Red and also concisely discussed on the acceptable daily intake, toxicology and extraction methods.

## Introduction

The additives used in food processing may be divided in two groups: (i) naturally occurring compounds or additives isolated from natural sources and (ii) synthetic chemicals that are widely applied in foods industry from many years ago. Natural color additives contain lower tinctorial strength as compared to synthetic colors because of more sensitive to light, temperature, oxygen, pH, color uniformity, low microbiological contamination, and relatively low production costs. Coloring used in food industry to improve the food appearance, flavor, taste, color, texture, nutritive value and conservation. Hence, synthetic food dyes stand out as one of the essential additive class for food industry in the conquest of markets (Ashfaq and Masud, 2002; Salem et al., 2009).

**FIGURE 1:**
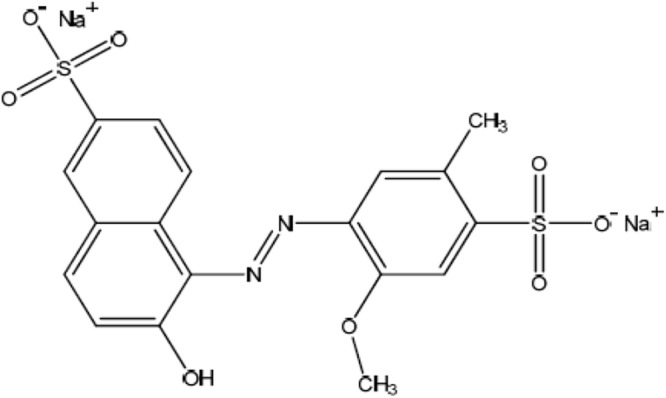
**Molecular structure of Allura Red AC (E 129)**.

Synthetic dyes are classified into azo dyes, triphenylmethane dyes, xanthene dyes, indigotine dyes, and quinoline dyes. Azo dyes contain azo group (-N = N-) as the chromophore in the molecular structure, which is largest group of color accounting more than half of global dyes production. One of the mostly used synthetic dyes in food industry is Allura Red (**Figure 1**), which could be found in many commercial foodstuffs, for example soft drinks, candies, ice cream and bakery products. Allura Red is an electrochemically active with irreversible reaction (Zhang et al., 2010). Previously, several researches have been reported regarding Allura Red toxicity and carcinogenic effects (Dinç et al., 2002; Kiseleva et al., 2003; Amate et al., 2010). Allura Red has potential behavioral effects on humans and animals; especially increase hyperactivity in children. Moreover, some studies have showed the presence of aromatic amine or amide functionalities in the chemical structures of the degradation products of Allura Red. Allura Red has absorbed to gastrointestinal and entered the bloodstream to associates with proteins during its transport and metabolism process (Combes and Haveland, 1982; Lok et al., 2006; McCann et al., 2007; Rebane et al., 2010; Gosetti et al., 2013). The excess usages of Allura Red in food and beverage products must be controlled.

In many countries, the uses of several food dyes including Allura Red has controlled or banned due to it toxicity. The lists of permitted synthetic dyes have different from each country, for examples, azorubine, quinoline yellow, and patent blue V are permitted in EU countries, but considered forbidden in Japan and USA. For the safety assessment, the Joint FAO/WHO Expert Committee on Food Additives (JECFA) and EU Scientific Committee for Food (SCF) established an acceptable daily intake (ADI) of Allura Red is 0–7 mg/kg/bw/day (European Food Safety Authority [EFSA], 2009). Due to the concern of human health, several analytical and advanced methods are developed for analyzing and quantifying of Allura Red. Thus, this review paper is emphasized the available of analytical and advanced methods for detection of Allura Red in food products, and also discussed on the ADI, toxicology and extraction methods.

## Food Colorant: Allura Red AC (E 129)

Natural and synthetic dyes are classified into soluble colorants. Natural colors are obtained from various food or natural materials, for example riboflavin (E 101), chlorophylls (E 140), carotenes (E 160a), betalain (E 162) or anthocyans (E 163). Natural colors are not precise stable, so it could be characterized by their specific physiological activity. Synthetic colors are originally manufactured from coal tar or purified oil products (Amchova et al., 2015). Synthetic food colors have high stability to light, oxygen, pH changes and relatively low cost as compared to natural color (Gómez, 2012). Synthetic food dyes are chemically synthesized which found wide compounds structures on their structural characteristics (Carmen, 2008). Azo dyes have found more than 3000 compounds in worldwide uses and accounted about 65% of the commercial dye in the market (Ahlström et al., 2005).

Codex Committee on Food Additives and Contaminants (CCFAC) formed an International Numbering System (INS) for food colorant to be identifying on the list of ingredients by a three-digit number. These given numbers have replaced by the specific name of the colorants that are so long due to complex chemical structure. Based on EU, a system of E numbers has implemented in order to identify all food additives. E number is composed of the letter E represented for Europe, followed by the INS three-digit number, for example Allura Red is E 129 (Amchova et al., 2015). Allura Red has been approved by European Union (EU) Register and listed in Annex I of Directive 94/36/EC. Allura Red most commonly used synonyms of Food Red No. 40 and Food, Drug and Cosmetics Red No. 40 (FD&C Red No. 40). Allura Red consisted of disodium 2-hydroxy-1-(2-methoxy-5-methyl-4-sulphonato-phenylazo)naphthalene-6-sulphonate and subsidiary coloring agents, with sodium chloride and sodium sulfate as the principal uncolored components (European Food Safety Authority [EFSA], 2012). Allura Red manufactured by coupling diazotized 5-amino-4-methoxy-2-toluenesulphonic acid with 6-hydroxy-2-naphthalene sulphonic acid. The molecular formula of Allura Red is C_18_H_14_N_2_Na_2_O_8_S_2_ (MW: 496.42 g/mol) and structural formula is shown in **Figure 1**. It is dark red in color and water-soluble powder or granules, but slightly soluble in 50% ethanol. The maximum absorption in water is 504 nm, at pH 7 (E_1__cm_^1%^ = 540). In order to replace Amaranth (E123), Allura Red AC was first time introduced in the US since 1980s and it had synthesized by the classical process of diazotization (Carmen, 2008). It has permitted to be used as a food additive in food products. However, it is not acceptable for use in animal feed because of the genotoxic effects (Ceyhan et al., 2013). USA Food and Drug Administration (FDA) have approved the uses of Allura Red in cosmetics, drugs, and food. Besides, Allura Red can be used in some tattoo inks. In US, Allura Red is commonly replacement used to Amaranth (Red 2) and Erythrosine (Red 3).

## Acceptable Daily Intake

The ADI is estimated of daily total intake of food colorants without any adverse effect on health. ADI is expressed as mg per kg of body weight (Duffus et al., 2009). To prevent excessive uses of Allura Red, some countries have legislated laws and regulations to limit the amounts permitted of Allura Red in food and drinks. Allura Red has been evaluated by the Joint FAO/WHO Expert Committee on Food Additives (JECFA) in 1980 and the EU SCF in 1984 and 1989. JECFA and SCF have established an ADI of Allura Red of 0–7 mg/kg/bw/day in food and beverages products (European Food Safety Authority [EFSA], 2009). Several countries such as EU, China, Japan, Australia, Brazil, and New Zealand have permitted the uses of Allura Red in food, drugs and cosmetics products less than ADI values. However, it has banned in USA, Denmark, Belgium, France, Switzerland, and India (European Food Safety Authority [EFSA], 2009). Accordingly, the uses of synthetic dyes in foodstuffs are strictly controlled by legislation throughout the world (EC, 1994 and GB2760-2011, 2011). Food industries have required to be listed on the package label to avoid the excess consumption of synthetic dyes. Food Safety Law of the People’s Republic of China has required the application of synthetic color additives to maintain in surveillance by the China Food and Drug Administration (CFDA) and listed in Direct GB 2760-2011 of the Ministry of Health because of legally used in food markets. According to the Direct GB 2760-2011, eleven synthetic colors are listed including Allura Red as certifiable food color additives that can be added in food products. The maximum amount has allowed the most synthetic food colors but not more than 100 mg kg^-1^ of colorants (GB2760-2007, 2007). The maximum permitted levels of the uses of Allura Red AC in beverages and foodstuffs are summarized in **Table 1** (European Food Safety Authority [EFSA], 2009).

**Table 1:** The Maximum Permitted Levels of Allura Red AC in beverages and foodstuffs (European Food Safety Authority [EFSA], 2009).

Product class	Types of food	Permitted level (mg L^-1^)
Soft drinks and other non-alcoholic beverages	Ready-to-drink cordials, vending machine concentrates, instant teas, bitter soda, bitter vino, liquid food supplements/dietary, integrators	10–100
Alcoholic beverages	Beers, ciders, fortified and aromatized wines, spirituous beverages, aromatized wine-product cocktails, fruit wines, cider and perry	Up to 200
Confectionery	Boiled sweets, toffees, caramels, gums, jellies, pastilles, licorice, chewing gum	50–300
Fine bakery wares	Biscuits, wafers, cakes, baking ingredients, preserves of red fruits, extruded or expanded savory snack products.	Up to 200
Soups	Complete formulae for weight control and nutritional supplements.	Up to 50
Meat products	Breakfast sausage with a minimum cereal content of 6%, luncheon meat	25
	Burger meat	20
	Luncheaon meat	25
Meat and fish analogs	Based on vegetable proteins	Up to 100
Fish products	Surimi	500
Cheese	Edible cheese rind	No maximum level specified
Special dietary foods	Liquid food supplements	Up to 100
Desserts	Blancmanges, custards, mousses, dry mixes, sauces	Up to 150
Snack foods	Other savory products and nuts	Up to 100
Sauces	Pickles, relishes, chutney, curry powder, tandoori	Up to 500

## Toxicology of Synthetic Food Colorants

Food adulterants are chemical or biological substances that are added into foods with intentionally reducing the manufacturing cost and artificially inflate food quality that caused illness to consumers (Eunice et al., 2010). Some synthetic food colorants can be toxic to aquatic organisms and carcinogenic effects on humans, particularly when they are extremely consumed (Ceyhan et al., 2013). Toxicological study of Allura Red was assessed by JECFA in 1980 and SCF in 1984 and 1989. Allura Red is able to reduce by azoreductase enzymes in intestinal bacteria and in liver cells with the release of aromatic amines to the organism that caused frequent headaches in adults while children often become distracted and hyperactive (Hawley and Buckley, 1976; Rafii et al., 1997; Huang et al., 2003). Feingold (1975) has initially claimed the detrimental effect of Allura Red to childhood behavior more than 30 years ago. Hyperactive is a pattern of behavior that showed substantial individual differences in the general population. Children probably found the above behavior pattern a large degree diagnosed with ADHD (Feingold, 1975; Overmeyer and Taylor, 1999; McCann et al., 2007). Recently, few studies have been published on the issue of Allura Red binding to human serum albumin (HSA) (Wang et al., 2014; Masone and Chanforan, 2015; Wu et al., 2015).

Scientific Opinion of the EFSA Panel has concluded that Allura Red may cause allergic reactions such as urticaria and asthma, especially when mixed with other synthetic color in food products. However, EFSA panel reported the ADI value was not changed due to the fact that current levels of use in the European population is far from reaching the official limit of 0.4–0.6 mg daily in the 95th percentile of the population (European Food Safety Authority [EFSA], 2009; Fallico et al., 2011). Several researchers have reported regarding its toxicity and carcinogenic effects on human health (Borzelleca et al., 1989; Jung et al., 1992). Sasaki et al. (2002) have studied the toxicity of several food additives, including Allura Red AC in mice. They found that there is no death observed at 2000 mg kg^-1^ Allura Red on four to five mice. So, it can be concluded that Allura Red has lower acute oral toxicity.

## Extraction Methods

Food colors first extracted from the food matrix and purified for the removal of the potential interfering coextractives for the analysis and quantitation. Some samples pretreatment are often required including defatting of meat products, dilution of sugars and gums in confectionery products, and then can be proceed for extraction procedure. Most extraction procedures are followed a common path involving in the release of desired analytes from their matrices, followed by removal of extraneous matter and a suitable extraction method (Thompson and Trenerry, 1995; Wu et al., 2013).

The supercritical fluid extraction (SFE) technology has advanced tremendously since its inception and is a good method in many food processing industries. Past two decades, SFE has been well received as a clean and environmentally friendly “green” processing technique and in some cases, an alternative to organic solvent-based extraction. The most recent advances of SFE applications in food science (Allura Red), natural products, by-product recovery, pharmaceutical and environmental sciences have been published in extensive reviews (Herrero et al., 2010). Supercritical fluid solvents are of interest in chemical processes both for their involvement in chemical reactions as well as their solvent effects that are influenced by pressure and temperature.

Solvent extraction known as liquid-liquid extraction (LLE) which has involved the separation of compounds based on their relative solubility with two different immiscible liquids (organic phase and water). The extraction of Allura Red is most common solvents used as like as water, ethanol, methanol, isopropyl alcohol, ammoniacal ethanol, ethyl acetate, ammonia, cyclohexane and tetra-n-butyl ammonium phosphate. Yoshioka and Ichihashi (2008) have used different solvents for the simultaneous extraction among forty food dyes in drinks and candies. They mentioned that the mixture of ammonia and ethanol (1:1, v/v) solutions have showed good extraction efficiency after ultra-sonication and evaporation of the sample. Similarly, Zou et al. (2013) have addressed the tri-mixtures of ethanol, ammonia and water (80:1:19, v/v/v), and found better extraction recoveries for seven dyes in animal feed and meat samples. Harp et al. (2013) have analyzed seven certified food colors in forty-four food products by liquid chromatography method using the ammonium hydroxide and methanol as extraction solvents. Khanavi et al. (2012) have established a green extraction procedure using non-organic solvents, which are ammonia (0.25%, v/v) and water for Allura Red extraction from food products and medicines.

Solid-phase extraction (SPE) known as absorption technique to separate food colorants by utilizing a variety of adsorption materials such as wool, powdered leather, cellulose, alumina, and polyamide powder. SPE commonly used because of simple procedure, rapid and able to treat large volume of samples free from contaminants with high recoveries. Recently, semi-micro adsorption cartridges containing reverse-phase bonded silica materials have widespread used. Typical sorbent for SPE include C_18_, while amino-functionalized low degrees of cross-linking magnetic polymer (NH_2_-LDC-MP), polyamide, gel permeation chromatography (GPC) and styrene-divinylbenzene polymer has good retention toward Allura Red (Soylak et al., 2011; Bonan et al., 2013; Chen et al., 2014; Tang et al., 2014). Different organic solvents have used in the analysis of Allura Red resulting in difficulty for selection of an appropriate solvent. The structure of analytical matrix and its components have played important role while selecting an appropriate solvent for extraction. Usually several solvents such as methanol, acetic acid, ethanol, acetone, ethyl acetate, tetra-n-butyl ammonium phosphate and others are more appropriately extracted of Allura Red. Tang et al. (2014) have used SPE for extraction among sixteen synthetic colorants in complex hotpot condiment with high oil content. The combination of methanol, acetone (1:1, v/v) and 2 mol L^-1^ carbamide solution containing 5% of ammonia in methanol showed good extraction efficiency while purified by a GPC column. Besides, Chen et al. (2014) have investigated the use of NH_2_-LDC-MP as a sorbent in SPE under magnetic field to enhance the extraction recoveries among seven synthetic food dyes by using water as an extraction solvent.

Enzymatic digestion of food samples are highly bound or associated with the food matrix. The combinations of enzyme-substrates are widely used including papain (protein digestion), lipase (lipids), phospholipase (phospholipid), amyloglucosidase (starch), pectinase (pectin), and cellulase (cellulose) (Rodriguez-Saona et al., 2001; Soltan and Shehata, 2012). It is one of most common method for extraction of Allura Red that included one-step extraction with membrane filter using water as diluents (Minioti et al., 2007; Gosetti et al., 2012). Other extraction methods such as dialysis, microwave-assisted extraction (MAE) and ultrasound-assisted extraction (UAE) are eco-friendly methods that frequently applied in food samples. Shen et al. (2014) have established new extraction method using two-phase solvent (methanol and acetone) and UAE that improved the extraction recovery of both hydrophilic and hydrophobic pigments for Allura Red extraction. Sun et al. (2013) have developed MAE extraction method for isolation of 21 synthetic colorants including Allura Red in meat by using methanol-acetic acid (95:5, v/v) as a solvent. In contrast, there are a few methods available using without extraction procedure before analyzing the level of Allura Red (Sorouraddin et al., 2011). The extraction methods are summarized in **Table 2**.

**Table 2:** Analytical techniques for determination of Allura Red in various food sample.

Methods	Matrix	Extraction procedure	Reference
HPLC	Crisps (potato chips), biscuits, drinks, desserts, sweets, ice cream, jam, cakes and miscellaneous snacks.	Solid, hard/jelly: 5.0 g was vortexed, sonicated and centrifuged for 5 min (2500 rpm). *Drinks or liquid samples:* 5.0 g dissolved and centrifuged for 10 min at 13,000 rpm and room temperature; 50 ml from the top clear supernatant was injected.	Lok et al., 2010
UHPLC–MS/ MS-DuoSpray	Beverage	Samples were filtered through 0.45 μm nylon filter and diluted 1/20 (v/v) in ultrapure water	Gosetti et al., 2012, 2013
MEKC	Confectionery and cordials	5 + 25 mL methanol-water (20:80, v/v), mixed, shaken vigorously for 5 min, centrifuged at 1000g (5 min), supernatant is filtered + 1 mL of 0.05 M tetra-n butylammonium phosphate solution and followed by SPE; eluted with methanol	Thompson and Trenerry, 1995)
LC-UV	Soft drinks, sugar and gelatin based confectionery	2.5 g + 25 mL H_2_O, sonicated (5 min) + heated (50°C for 30 min) + 25 mL acetate buffer solution (0.2 mol/L, pH 5.0) and filtered. Then, 10 mL of above solution + 350 μL RTIL + manually shaken (30 times in 20 s) + centrifuge (3500 rpm, 8 min), discard supernatant and residue IL’s is mixed with methanol to get a volume of about 300 μL enriched analytes.	Wu et al., 2015
LC-PDA	Fruit flavored drinks, alcoholic drinks, jams, sugar confectionery and sweets	10 mL or g sample + 40 mL H_2_O; degassed (15 min) and filtered through a folded filter paper to collect the filtrate and filtered through 0.45 μm syringe filters before injection.	Minioti et al., 2007
UFLC-MS/MS-ESI	Wine and soft drinks	*Magnetic dispersive solid-phase extraction (M-dSPE):*1.0 mL of sample in an open evaporating dish, evaporated to dryness in a water bath at 80°C. Residue was re-dissolved with pure water (pH 9.0 adjusted with ammonia 0.5 mol/L) and transferred to a polypropylene centrifuge tube (2.0 mL) containing 15.0 mg of NH_2_-LDC-MP, vortexed for 1.0 min, adsorbed NH_2_-LDC-MP was isolated under magnetic field and 0.5 mL aliquot of the supernatant was filtered using a 0.22 μm membrane prior to its injection.	Chen et al., 2014
LC-DAD	Hotpot condiment	5 g sample + 20 mL of methanol-acetone (1:1,v/v) and heated at 60°C on water bath for 20 min; vortexed (2 min) and centrifuged at 5000 rpm (5 min), repeat the extraction with 20 mL of methanol-acetone (1:1, v/v), 2 × 15 mL of 2 mol/L carbamide solution containing 5% ammonia (dissolved in methanol). Two extracts were combined and concentrated.	Tang et al., 2014
LC-DAD	Foodstuffs and beverages	*Solid food matrices*: 4 g + 20 mL ethanol-water (1:1, v/v), homogenized, sonicated (10 min), shaken (1 h) and centrifuged at 15,000 rpm for 10 min at 0°C; solid residue is collected, extracted with 20 mL of solvents mixture; pH adjusted to 2 with H_3_PO_4_ drop wise followed by extraction with SPE polyamide cartridge using 4.5 mL of methanol/1% ammonia solution (1:1, v/v) as eluent. *Beverages:* Degassed sample is diluted (1:1, v/v) with deionized water and centrifuged at 15,000 rpm for 10 min at room temperature.	Bonan et al., 2013
UV-visible spectrophotometer	Water	25 mL sample, filtered through 0.45 μm; pH adjusted to 4.0 and extracted with 3 mL ACN as eluent by solid phase extraction glass column (MCI GEL CHP20P resin).	Soylak et al., 2011
LC-PDA	Food stuffs	*Ultrasound-assisted solvent extraction:* 0.50 mL/0.20 g in 15 mL test tube + 1 mL methanol. Probe sonicator is immersed (15 min) into the sample mixture to perform ultrasound-assisted solvent extraction. Centrifuged to collect supernatant layer in another test tube. Repeat 3x methanol extractions. Combine all supernatants; precipitated residue + extracted with 3x of 1 mL acetone. Combine acetone layers and evaporated using centrifuge vacuum evaporator; methanol supernatant + dried acetone extract, followed by evaporation. Final dried extract + 2 mL methanol, filtered (0.45 lm micro-porous film) and transferred to vial.	Shen et al., 2014
LC-PDA	Dairy products and fatty foods	Automatic solid-phase extraction system using cotton and RP-C_18_ columns for the sequential retention of synthetic colorants and natural colorants, respectively.	González et al., 2003
UHPLC-DAD	Meat	*Microwave assisted extraction*: 10 g homogenized samples + 1.5 mL of 10 μg/mL color standards of each analyte + homogenized (5 min); extracted with 15 mL of methanol:water (95:5, v/v) using microwave extraction under 100 psi, and 50 W at 80°C for 5 min; centrifuged (15,000 rpm, 3 min at 4°C), supernatant is collected and processed for SPE using C_18_column and eluted with 4 mL of methanol-acetic acid (95:5, v/v). The collected eluates were evaporated to dryness at 50°C and dry residues were reconstituted with 0.5 mL methanol, and filtered through 0.22 μm membrane filter before analysis.	Sun et al., 2013
LC-DAD	Drinks, syrups and candies	*Drinks and syrups*: 10 g sample + degassed in case of beverages (5 min)/evaporate ((if alcoholic drink) and make up the volume with H_2_O) + 10 mL of H_2_O; mixed and adjusted to pH 3 with 6% acetic acid. *Candies and marsh mallows*:10 g powdered sample + 50 mL H_2_O; dissolved by heating and pH adjusted to 3 with 6% acetic acid *Extraction:* Polyamide column separation was employed for the extraction. 15 mL of 1% ammonia solution/ethanol (1:1, v/v) as eluent, eluate collected; filtered, neutralized with 6% acetic acid and dried. The residue obtained was reconstituted with 2 mL of methanol/water (1:1, v/v).	Yoshioka and Ichihashi, 2008
LC-DAD-ESI/MS/MS	Animal feed and meat	2 g of feed or homogenized chicken meat mixed with series of 7-dye mixed standard solutions and extracted with 10 mL of C_2_H_5_OH:NH_3_:H_2_O (80:1:19, v/v/v) and centrifuged at 12,000 rpm for 10 min at 4°C. Collect the supernatant and dried at 50°C under nitrogen stream. Reconstitute the residue with 0.5 mL of C_2_H_5_OH:NH_3_:H_2_O (80:1:19, v/v/v).	Zou et al., 2013
LC-DAD	Fish roe	5 g + 20 mL aqueous ammonia, sonicated (10 min), centrifuged (3500 *g*); aqueous layer was collected. Repeat the extraction until the aqueous layer becomes colorless. Combine the aqueous layers and is defatted by 3 × 10 mL n-hexane, pH adjusted to 2 (1 M HCl) + 2 g polyamide, stirred at 40°C (20 min), filtered and repeat the same until solution becomes colorless. Colorants were desorbed using (1/9, v/v) of ammonia solution (25%) and methanol.	Kirschbaum et al., 2006
LC-PDA	44 food products	*Beverages, frozen treats, powder mixes, and gelatin products:* 5 g sample (carbonated drinks were sonicated to flatness before weighing.) + 100 μL of 10% (v/v) aq. NH_4_OH, make up the volume (10 mL) with methanol and filtered. Powder mixes 0.1 g sample dissolved in a 50% methanol solution containing ∼100 μL of 10% aq NH4OH with sonication and followed by filtration. Gelatin product 0.5 g sample + 50% methanol solution containing ∼100 μL of 10% aq NH_4_OH and filtered. *Candies, icings, jellies, spices, dressings, sauces, baked goods, and dairy products:* 5 g homogenized sample + 10 mL of 7:3 methanol/10% aq NH4OH (v/v), vortexed for 1 min, sonicated for 1 h at 38°C with periodic shaking, and centrifuged for 5 min at 8500 rpm. aq extract was transferred into a clean 50 mL centrifuge tube. The remaining product is washed by adding 10 mL of 7:3 methanol/10% aq NH_4_OH, sonicated with heat for 5 min, and centrifuged for 5 min at 8500 rpm Mix aqueous extract with the previously collected extract. Repeat the washing step two more times and centrifuged for 5 min at 8500 rpm if necessary by adding 2 mL of n-hexane. To the lower aq extract ∼20 μL of concentrated acetic acid is added, filtered and transferred into an LC vial for analysis.	Harp et al., 2013
LC-PDA	47 food products	*Beverages and powder mixes, fruit jellies, and hard candies:* 25–30 mL (beverages) or 5–10 g (powder mixes and fruit jellies, or two or more units of hard candy) + 50 mL water, stirred and gentle heat (∼60°C) if necessary and filtered. *Cookies, cereal, wafers, chips, noodles, and gummy candies:* 15 g powdered sample + 200 mL of 25% glacial acetic acid + heated (∼60°C) vacuum filtered and centrifuged (10 min at 4500 rpm). Collect the supernatant, filtered and concentrated to no less than 50 mL by applying gentle heat (∼60°C) with constant stirring followed by isolation cleanup and recovery steps using SPE *Isolation and cleaning step*: 5 mL (1% acetic acid in methanol) + 2-5 mL of sample extract + 5 mL of water. *Recovery step*: 2 mL (10% ammonium hydroxide in methanol), eluted with 2 mL of methanol and evaporated to dryness + 2 mL of 1:1 (v/v) methanol/ water to reconstitute the analytes.	Harp et al., 2012
LC-UV	Food stuff and medicines	*Drink and syrup*: 25 mL sample + 25 mL deionized water. *Candy and jelly*: 5 g (candy powder or jelly) dissolved in 25 mL flask and make up the volume with deionized distilled water. *Chocolate in crisp sugar shell*: 5g powder + 20 mL NH_3_ (0.25% v/v), shaken slowly to remove dye and then separate the uncolored residue *Blowgun (Pofak)*: 5 g powder + 20 mL NH_3_ (0.25% v/v); extracted using 20 mL NH_3_ (0.25%, v/v) with ultra-sonicated and filtered. Repeat the procedure to dissolve remaining dye from texture and the mix the solutions.	Khanavi et al., 2012
UV-visible spectrophotometer	Granulated drinks	*Zero crossing derivative spectrometry (ZCDS), ratio derivative spectrophotometry (RDS) and compensation technique (CT)*: 2.0 g powdered sample dissolved in 50 mL distilled water, stirred for 5 min with magnetic stirrer and the solution is further diluted 3:10 (v/v) with water. *Derivative-difference spectrophotometry (DDS):* 2.0 g powdered sample dissolved in 50 mL distilled water, stirred for 5 min with magnetic stirrer and the solution is further diluted 3:10 (v/v) with 0.1 M HCI and 0.1 M NaOH separately. No further extraction or evaporation step is required.	Turak and Ozgur, 2013
MEEKC	Food products	Rarely required (e.g., SPE).	Huang et al., 2005
CE-UV/vis	Alcoholic beverages	Samples degassed by mechanical agitation and filtered through 0.45 μm pore membrane filter.	Prado et al., 2006
HLA/GO	Soft drinks	Samples 2.0 mL in 100 mL water; heated (10 min); centrifuged for 30 min at 3500 rpm and filtered.	Al-Degs, 2009
LC-PDA	Soft drinks	Samples were filtered and degassed (if carbonated), pH adjusted to 6.5 with 10% NaOH.	Jurcovan and Diacu, 2014
Reversed-phase TLC	Food products	Methanol-acetonitrile-5.0% aqueous sodium sulfate	Oka et al., 1994
Capillary electrophoresis	Beverages	25 m*M* sodium phosphate buffer and 25 m*M* sodium borate buffer (1:1) (pH 8.0) containing 10 m*M* sodium dodecyl sulfate (SDS).	Suzuki et al., 1994

## Analytical Techniques for Determination of Allura Red AC

Food coloring is one of the food adulterants which chemicals substances that intentionally added to food in order to improve customer’s perceptions of food. The presences of Allura Red in potentially interfering compounds are difficulty to identify by using analytical methods. For Allura Red, several analytical methods have developed such as voltammetry, polarography, spectrophotometry, mass spectrometry, capillary electrophoresis (CE), ion chromatography, thin layer chromatography, high-performance liquid chromatography (HPLC), liquid chromatography-mass spectrometry (LC-MS), and liquid chromatography-tandem mass spectrometry (LC-MS/MS).

### High-Performance Liquid Chromatography (HPLC)

High-performance liquid chromatography becomes the major analytical method for determination of synthetic coloring materials in foodstuffs. The most widely used separation modes are ion exchange and reverse-phase. Other method used for separation, qualitative and quantitative determination of synthetic food dyes based on high performance liquid chromatography. The basis of separation has two phases; stationary phase and mobile phase. Dyes have different adsorption affinity to stationary phase. It has appeared from differences of their mass, structural space and presence of functional groups in each dye’s molecule. A wide range of liquid chromatography based techniques have analyzed for the detection of azo dyes, most of them are coupled with UV-Vis, PDA or MS detectors. The HPLC technique has reversed phase high performance liquid chromatography (RP-HPLC) and ion-pair high performance liquid chromatography (HPLC-IP).

In RP-HPLC system, the mobile phase has stronger polarity such as tetrahydrofuran, acetonitrile, methanol and water, while stationary phase is slightly polar or non-polar (Witkiewicz, 2000). Appropriate conditions are allowed for analyzing the most of food dyes. Ionized samples must have possibility to form neutral molecules. The most important characteristics into consideration during selection of hydrophobic properties are tested and presence the molecules with acidic groups. Hydrophobicity of azo dyes is the largest group as compare to other. Ion pair reverse-phase chromatography (IP-RP-HPLC) consisted in adding hydrophobic ionic substance to the mobile phase. It could be quaternary ammonium cation, alkilo- or arylsulfoniumanion. As a result of the reaction between sample and eluent neutral ionic pairs are formed and separated chromatographically in the reversed phase system. Another way is preparing of sample, which enables the conducting of analysis on ionic exchanger or modification of mobile phase that provides to obtain the ion-exchanger (Witkiewicz, 2000).

In contrast, HPLC combined with diode array detection (HPLC-DAD) is very popular for qualitative and quantitative determination with excellent precision, accuracy and lower cost, which can be more practical and economical in detecting non-illicit additives such as food colorants. Qi et al. (2015) developed an efficient, fast and sensitive method for determination of 11 synthetic dyes including Allura red, in flour and meat foodstuffs using HPLC coupled with DAD and MS/MS. The color additives are extracted with ammonia-methanol for further purified with SPE procedure using Strata-AW column in order to reduce matrix interference. The proposed method is intended for a comprehensive survey of color additives in foods. HPLC-MS/MS method is used for further confirmation of the results. Validation data showed good recoveries in the range of 75.2–113.8%, with relative standard deviations less than 15%. The proposed method has proved more suitable for the routine monitoring of eleven synthetic color additives due to its sensitivity, fast and low cost. Li et al. (2015) developed HPLC-DAD combined with ESI-IT-TOF/MS in positive and negative ion modes for identification and quantification among 34 water-soluble synthetic dyes in foodstuff. Under optimal condition, the averages LOD of dyes were found between 0.01 and 0.05 μg mL^-1^. The recoveries and RSD range between 76.1–105.0% and 1.4–6.4%, respectively. Karanikolopoulos et al. (2015) developed the protocol based on RP-HPLC/DAD for the analysis of Allura Red in complex food matrices presenting high protein and fat content. The issue of high fat content matrices addressed; it was needed an additional defatting step in the procedure. The proposed method showed high precision and accuracy of detection in other complex food matrices. Other method developed by Kong et al. (2015) based on freeze method for deproteinization coupling with the chitosan purification process in protein-rich samples. Chitosan used for the purification after deproteinization as compared with the traditional technique. Under optimum conditions, the method showed good linearity between 0.6 and 10 mg kg^-1^, with LOD between 0.1 and 0.4 mg kg^-1^.

Bazregar et al. (2015) established a method based on the electro-kinetic migration of ionized compounds by the application of an electrical potential difference. Efficient extraction technique is used with a sub-microliter organic solvent consumption termed as in-tube electro-membrane extraction (IEME). The result showed high extraction yield recoveries and the consumption of the organic solvents are less. IEME-HPLC-UV showed a good linearity in the range of 1.00–800 ng mL^-1^, with LOD of 0.3-1.0 ng mL^-1^. Tsai et al. (2015) have simultaneously determined among 20 synthetic dyes including Allura Red by using LC-MS/MS method. The linearity and recoveries are observed at the concentration range of 0.10–200 μg kg^-1^ and more than 90% for all dyes. Chen et al. (2014) developed a sensitive method based on the use of magnetic dispersive solid-phase extraction (M-dSPE) procedure combine with ultra-fast liquid chromatography-tandem quadrupole mass spectrometry (UFLC-MS/MS). The obtained results showed higher extraction capacity of NH_2_-LDC-MP with recoveries between 84.0 and 116.2%, with limit of quantification (LOQs) for the seven synthetic pigments are of 1.51 for wines and 5.0 μg L^-1^ for soft drinks. The developed M-dSPE UFLC-MS/MS confirmed that the NH_2_-LDC-MP is a kind of high effective M-dSPE materials for the pigments analyses.

Jurcovan and Diacu (2014) developed a simple method for the simultaneous measurement of Allura Red and Ponceau 4R in soft drinks by employing water and acetonitrile as a mobile phase. Bonan et al. (2013) proposed the simultaneous analysis of red and yellow dyes by using HPLC-DAD in solid food matrices and beverages. A water-alcohol mixture, cleaned up on a polyamide SPE cartridge and eluted with basic methanol solution, extracts the food samples. The method is successfully validated according to Regulation (2004/882/CE) and could be applied to a concentration range between 5 and 300 mg kg^-1^ (5–100 mg l^-1^ for drinks) depending on the dyes. Tang et al. (2014) have determined among 16 synthetic colorants in hotpot condiment by HPLC. Based on results, a good linear relationship between peak areas and the concentrations of the synthetic colorants are obtained with LOD of 1–3 μg kg^-1^. The proposed method is more sensitive and reliable that can be used for simultaneously determined among eight lipid-soluble and eight water-soluble colorants in hotpot condiment.

### Mass Spectrometry and Spectrophotometry

Various spectrometry techniques are available for the analysis of Allura Red including the measurements at ultraviolet and visible wavelengths. Spectrometry is suitable for quantitative analysis of food dyes in different food matrices. Spectrometry frequently applied for determination of Allura Red because of high values of molar absorption. Spectrometry shows low instrumentation cost and does not require any expert skill manpower. The distinguishing features of the spectra obtained for single color is significantly affected by the adjustment of pH of the solution with acid or alkali; characterized by shifts in absorption wavelength maxima and intensities. María et al. (2007) have used time flight mass spectrometry (TOF-MS) instruments that represent a valuable tool for screening of target and non-target compounds in food products. Accurate mass measurements along with specific retention times can be detected highly reliable target species, avoiding isobaric interferences in complex samples. Moreover, a mass spectrometry combine with an ESI (or APCI) source and an ion trap analyzer linked to a TOF mass analyzer (ESI/APCI-IT-TOF/MS) that able to provide multistage tandem spectra with accurate masses. This feature makes IT-TOF/MS useful for identifying target dyes and non-target dyes in foodstuffs. Holčapek et al. (2007) investigated various functional groups of synthetic dyes that could affect their fragmentation behavior in the sources of ESI and APCI. Currently, there are interested in the fragmentation mechanism of synthetic food dyes using ESI-IT-TOF/MS*^n^* in positive and negative ion modes.

Spectrophotometric method is simple, direct, rapid and versatile. Turak and Ozgur (2013) simultaneously determined Allura Red and Ponceau 4R in drinks with four derivative spectrophotometric methods as compared to the results with those of HPLC method. Soylak et al. (2011) developed a simple method with appreciable precision and low analytical cost the spectrophotometric determination of Allura Red in water samples by sensitive SPE procedure extraction on a glass column containing MCI GEL CHP20P resin. A new application of bulk liquid membrane (BLM) with second-order calibration based on the bilinear least squares/residual bilinearization (BLLS/RBL) algorithm as a novel method for simultaneous removal and quantification of Allura Red and Sunset Yellow which model compounds in soft drinks and food samples (Khani et al., 2015). The proposed method was validated by comparison with a reference method based on HPLC-UV and found no significant differences between the reference values and the obtained values. El-Sheikh and Al-Degs (2013) simultaneously quantified three common synthetic food color including Allura Red in powdered soft drinks by employing a combination of absorbance spectra-pH data matrices and multivariate processing of the generated second-order data. They used PARAFAC and bilinear least squares/residual bilinearization BLLS/RBL that applied for deconvolution of trilinear data to get spectral and concentration profiles of the dyes as a function of pH. The comparison of chemometric results with those obtained by standard chromatographic technique has proven that the former protocol is a reasonable accuracy with satisfied recoveries study.

### Capillary Electrophoresis

Capillary electrophoresis has been widely used for the analysis of Allura Red. It is an electrophoretic method to perform in a capillary tube for analysis and efficient separation of both small and large molecules. The separations of Allura Red are influenced by buffer composition, pH, and additives such as cyclodextrins. CE analysis showed rapid and economic as compared to the conventional electrophoresis and chromatography. Modern CE is driven by the production of low cost narrow-bore capillaries for gas chromatography (GC) and high sensitive on-line detection systems for HPLC. Besides, CE has a wide range of separation modes which including capillary zone electrophoresis, micellar electrokinetic capillary chromatography (MEKC), and capillary isotachophoresis etc., to complete efficient separations using high voltage (Xu, 1996). Thompson and Trenerry (1995) developed a rapid and economical method for determination of ten commonly used azo dyes including Allura Red in confectionary and cordial by MEKC. Similarly, Huang et al. (2005) established a microemulsion electrokinetic chromatography (MEEKC) method for the analysis of eight food colorants using a microemulsion solution. Prado et al. (2006) analyzed eleven synthetic food dyes in alcoholic beverages without any sample pre-treatment using CE-UV/Vis with excellent result.

### Thin-Layer Chromatography (TLC)

Thin-layer chromatography (TLC) is a simple, economic and most appropriate chromatographic technique for qualitative analysis of the mixtures of analytes. TLC systems for the separations of food dyes are fairly widespread; however, it is gradually being superseded by HPLC. Besides, one of the difficulties is facing an appropriate mobile phase and stationary phase, on which dyes are applied. A few TLC methods for the analysis of synthetic azo dyes have reported by Soponar et al. (2008). Kucharska and Grabka (2010) have reviewed various sample preparation techniques and chromatographic conditions for the analysis of synthetic dyes in different food samples by TLC and HPLC. de Andrade et al. (2014) have analyzed synthetic food dyes in soft drinks using SPE technique and analytes eluted by a mixture of isopropyl alcohol and ammonium hydroxide as the mobile phase.

## Advanced Techniques for Determination of Allura Red AC

### Electrochemical Sensors

**FIGURE 2:**
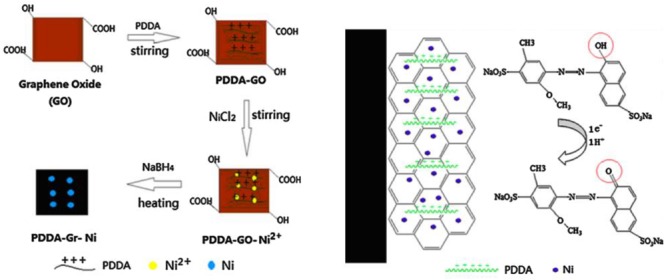
**The proposed scheme of the formation of PDDA-Gr-Ni composite and mechanism for the electrochemical process of Allura Red (Yu et al., 2016)**.

Electrochemical sensors have been widely applied for the analysis of Allura Red in foods due to fast response, low cost, simple operation procedure, required small amount and high sensitivity (Ahammad et al., 2011; Shaikh et al., 2013). It is feasible to miniaturize instrument for on-site detection. Recently, Yu et al. (2016) fabricated a sensitive and facile electrochemical sensor based on composite of poly(diallyldimethy- lammonium chloride) functionalized graphene with nickel nanoparticles on glassy carbon electrode (PDDA-Gr-Ni/GCE) to determine Allura Red. PDDA-Gr-Ni/GCE showed excellent mechanical strength, large specific surface area and high thermal and electric conductivity. The peak current of Allura Red exhibit remarkably increased on PDDA-Gr-Ni/GCE because of synergistic effect on the large surface area and improved electron transfer efficiency of the nanomaterial. The electrochemcial process of Allura Red on surface of PDDA-Gr-Ni/GCE involves one electron and one proton transfer as illustrated in **Figure 2**, which revealing that the oxidation of Allura Red is irreversible. Under optimum conditions, the limit of detection (LOD) found of 8.0 nmol L^-1^. Wang and Zhao (2015) developed an electrochemical sensor based on the modification of GCE with multi-walled carbon nanotubes in ionic liquid-graphene oxides (IL-GO-MWCNT/CGE). Different concentration of Allura Red was detected in the ranges of 8.0 × 10^-10^ – 5.0 × 10^-7^ mol L^-1^, with LOD value of 5.0 × 10^-10^ mol L^-1^ (S/N = 3). Rodríguez et al. (2015) studied an antimony film electrode prepared on-line and installed as part of a sequential injection system for determination of azo dyes in food samples. The influence of several flow variables is evaluated using a central composite design. The LOD was found of 0.3 μM with relative standard deviation (RSD) more than 5.0%. Cheng et al. (2015) have prepared a series of porous carbon (PC) using CaCO_3_ nanoparticles as the hard template and starch as the carbon precursor to determine azo dyes including Allura Red. The LOD was determined on the range of 1.4–1.7 μg L^-1^.

Zhang et al. (2010) constructed multiwall carbon nanotubes (MWCNTs) as sensing film to modified the surface of GCE due to have large surface area and high accumulation efficiency. The MWCNTs/CGE has enhanced the oxidation signals of Allura Red and Ponceau 4R Red during electrochemical reaction process. The MWNTs film sensor possesses high sensitivity to Allura Red and Ponceau 4R, and LOD found as low as 15 and 25 μg L^-1^, respectively. The proposed method was successfully used to detect Allura Red and Ponceau 4R in different beverages with satisfactory results. Silva et al. (2007) developed a polyallylamine modified tubular GCE for determination of three type azo dyes including Allura Red in several food samples by using Square wave voltammetry (SWV). The LOD of Allura Red value was obtained 1.4 μmol L^-1^. Alghamdi (2005) also determined of Allura Red by using SWV technique with hanging mercury drop electrode (HMDE). Adsorptive voltammetric method is detected in the concentration ranges from 2.5 × 10^-8^ – 2.0 × 10^-7^ mol L^-1^ (*r^2^* = 0.998), with LOD of 8.5 × 10^-9^ mol L^-1^. The proposed method has successfully applied for determination of Allura Red dye in commercially available candy and a soft drink. In conclusion, electrochemical techniques have several advantages such as high sensitivity, rapid, simplicity and promising the enhanced material for detection of Allura Red with food safety level.

### Differential Pulsed Polarography (DPP)

Differential pulsed polarography and differential pulse adsorptive stripping voltammetry are used for the estimation with different concentrations in food dye matrices. The addition of gelatin has advantageous in the partial identification and determination of food colors due to its pronounced effects on the measured peak currents. Combeau et al. (2002) addressed DPP that possible to differentiate Azorubin, Ponceau 4R and Allura red from the natural dyes providing from fruits. They are used different electrolytes that are tested such as potassium chloride, which is a classical supporting electrolyte, citric acid which is one of the components of the soft beverages, sodium citrate and a phosphate buffer. Chanlon et al. (2005) developed a sensitive method that could be easily distinguished from the natural dyes using DPP in syrups, soda, and sweets. The influence of the pH on the intensities and the potentials of the peaks are found between pH 3 and 11, and acidic or strong basic media but seemed not convenient. This method is successfully applied for monitoring food dyes in the commercial soft drinks and sweets with satisfactory results.

### Molecularly Imprinted Polymers (MIPs)

Molecularly imprinted polymers (MIPs) are biomimetic synthetic receptors possessing specific cavities designed for target molecule. A templating process has produced at the molecular level by co-polymerization of functional and cross-linking monomers; MIPs are capable of recognizing and binding target molecules with specificities and affinities comparable to those of natural receptors (Alexander et al., 2006; Bui and Haupt, 2010). It is extensively used in many fields including the SPE and chemo-/bio-sensors due to easy preparation, good stability, and highly specificity. The molecular imprinting coupling to the SPE has become an ideal technique for the extraction and enrichment of the trace analysis from the complicated food matrixes (Wang et al., 2009; Tan et al., 2012). MIP-functionalized magnetic composites have become a hotspot issue owing to the bi-functional property of the target molecule and the rapid magnetic response (Hu et al., 2013). MIPs have commonly showed excellent recognition selectivity and higher adsorption ability for determination of synthetic food dyes.

## Conclusion

Food additives are substances added to food to preserve flavor or enhance its taste, nutritional values and appearance. However, the various effects of food additives and preservatives on human as a result of the indiscriminate uses by food producers and food consumers. Many effects like food allergies, food intolerance, cancer, multiple sclerosis, attention deficit hyperactivity disorder (ADHD), brain damage, nausea, cardiac disease among others have been reported. European Food Safety Authority [EFSA] (2009) has revised ADI values and re-evaluated safety concerns of all synthetic dyes since 2008. The developments of analytical and advance methods with high sensitive and selective are needed to monitor of Allura Red in various food matrices. Sample preparation procedures have involved varying extraction techniques including leaching and SFE, solvent extraction, enzymatic digestion, membrane filtration and solid phase extraction techniques. Most of these analytical methods are actual complicated pre-concentration, time-consuming steps and high-cost instruments limit the application of existing methods. Therefore, these methods are unsuited for rapid and efficient or point-of-use foods dyes determination. In contrast, determination of Allura Red in foodstuffs based on the advanced methods (electrochemical sensor, differential pulsed polarography, MIPs) can offer various advantageous such as simple operation procedure, faster analysis, saving time, high sensitivity and selectivity, real time detection and low detection limit. Future study regarding the unknown pharmacological mechanisms of food colorants need to be highlighting. Moreover, systematic research should perform as like as elucidate pharmacological, neurodevelopmental and other effects that various colorants or their mixtures may have. We can suggest to the Government pass a law refusing permission for the food industries to add unauthorized toxic agents into our daily foods and beverages products. We need to protect the health of our population of young children, youths, adolescents and adults, as well as the health of our future generation because a healthy nation is a wealthy nation.

## Author Contributions

All authors listed, have made substantial, direct and intellectual contribution to the work, and approved it for publication.

## Conflict of Interest Statement

The authors declare that the research was conducted in the absence of any commercial or financial relationships that could be construed as a potential conflict of interest.

The reviewer PAP and handling Editor declared their shared affiliation and the handling Editor states that the process nevertheless met the standards of a fair and objective review.
